# Evaluation of Continuing Professional Development for Physicians – Time for Change: A Scoping Review

**DOI:** 10.5334/pme.838

**Published:** 2023-06-02

**Authors:** Shera Hosseini, Louise Allen, Faran Khalid, Donny Li, Elizabeth Stellrecht, Michelle Howard, Teresa M. Chan

**Affiliations:** 1 McMaster University, Faculty of Health Sciences, Department of Family Medicine, Canada; 2 Monash Center for Professional Development and Monash Online Education, Australia; 3 Michael G. DeGroote School of Medicine, Hamilton, Ontario, Canada; 4 Faculty of Health Sciences, McMaster University, Hamilton, Canada; 5 Division of Supportive and Palliative Care, William Osler Health System, Brampton, Canada; 6 Department of Research, Humber River Hospital, Toronto, Canada; 7 Head of Health Sciences Library Services, University Libraries, University at Buffalo, Buffalo, NY, US; 8 Department of Family Medicine, McMaster University, 100 Main S. W., Hamilton ON, L8P 1H6, Canada; 9 Division of Emergency Medicine, Department of Medicine, McMaster University, Hamilton, Canada; 10 Continuing Professional Development, Faculty of Health Sciences, McMaster University, Canada; 11 McMaster Education Research, Innovation, and Theory (MERIT) program, Faculty of Health Sciences, McMaster University Hamilton, Canada

## Abstract

**Introduction:**

Evaluation of education interventions is essential for continuous improvement as it provides insights into how and why outcomes occur. Specifically, for physicians’ continuing professional development (CPD) programs, which aim to upskill physicians in a range of practice-essential domains, evaluations are crucial to assure physicians’ continuous development, enhanced patient care and safety. However, evaluations of health professions education (HPE) interventions tend to be outcomes focused, failing to capture how and why outcomes occur. This scoping review aimed to identify evaluation techniques used to evaluate CPD programs for physicians, and to determine how these techniques are being implemented as well as the their quality.

**Methods:**

We searched PubMed, Embase, Web of Science, among others for English publications on evaluation of CPD programs for physicians, in the past decade. We used a data charting template to extract study details regarding the evaluation techniques and produced a checklist to assess the quality of the evaluations.

**Results:**

101 studies were included; of which 91 studies did not use an evaluation framework. Our findings revealed shortcomings in the evaluations of CPD programs including lack of attention to: intervention processes; unintended outcomes and contextual factors; use of theory; evaluation framework use; and rationale for chosen evaluation method.

**Discussion:**

Our findings highlighted major gaps in the evaluation techniques employed in physicians’ CPD. Attention needs to be paid to evaluating both program processes and outcomes to illuminate how and why impacts are or are not occurring.

## Introduction

Continuing professional development (CPD) refers to the activities that physicians undertake to maintain, update, and enhance their attitudes, knowledge, skills, performance, and relationships required to be effective clinicians, educators, researchers and leaders [1,2]. The central goal of CPD is to enhance patient care and safety through the maintenance of evidence-based practice, the implementation of new clinical practices, and the “de-implementation” of non-evidence-based, outmoded practices [3]. Thus, evaluation of CPD is critical for determining the outcomes and effectiveness of various CPD activities as well understanding how and why certain outcomes occur.

In health professions education (HPE), educators mostly use outcome evaluation when evaluating interventions [4]. Outcome evaluation approaches look to answer the question ‘Did it work?’ focusing on the impacts of an intervention [4]. While it is important to understand the outcomes of an intervention, outcome evaluation approaches do not explain how and why outcomes are occurring, or what else is happening, as they fail to explore the processes that lead to outcomes, acknowledge the complexity of educational interventions, and consider unintended outcomes. Alternately, program evaluation refers to evaluation of design and implementation in addition to the outcomes and allows for understanding how and why programs work. There are a range of program evaluation models in HPE and healthcare broadly (e.g., realist evaluation; contribution analysis; context, input, process, product (CIPP) evaluation). Educators have called for a shift from an outcome evaluation approach to a program evaluation approach to shed light on the mechanistic processes of why and how programs work (or not) [5,6,7]. Additionally, it is unclear what program evaluation strategies are best applied to single CPD events or which might be better applied to an entire CPD providing unit (e.g., an Office of CPD).

Current reviews on CPD program effectiveness focus on reporting outcomes, with little consideration given to the implementation of evaluation models being used, and the quality of evaluations being conducted. For example, one review investigated the impacts of CPD with no attention to evaluation quality [8], while another focused on the use of outcome and program evaluation models and advocated for increased use of program evaluation models, as opposed to focusing on evaluation quality [4]. Thus, this study aims to conduct a scoping review to answer the following questions:

- What evaluation techniques are being used to evaluate CPD programs for physicians, and how are these techniques being implemented?
- What is the quality of evaluations of CPD programs for physicians?

## Methods

Our scoping review adhered to guidelines posed by Arksey and O’Malley [9].

### Search Strategy

A health sciences librarian (ES) conducted an electronic search with retrieval limited to English-language publications published from January 1, 2010 to March 24, 2021. We chose this time frame to include the most recent patterns of CPD program evaluation in an evolving field. ES searched PubMed, Embase via Elsevier, Web of Science Core Collection, Education Source via EBSCOhost, and APA PsycINFO via EBSCOhost using a combination of keywords and subject headings representing different medical specialties, continuing medical education, and program evaluation, ProQuest Dissertations and Theses Global was also searched. ES originally built the search in PubMed and translated it for the other databases (See Appendix 1 for the PubMed search). Citations were exported to Covidence for study management and screening [10].

### Study selection

Two reviewers (DL, FK) independently completed article screening against the study eligibility criteria at the title and abstract and performed full-text screening at the full-text level. Any disagreements were resolved through discussions. Studies that included an evaluation of structured CPD training or intervention (with various foci: e.g., teaching, mentorship, leadership, skill development) for physicians were included. Studies were excluded if they were: abstracts, commentaries, reviews, conference posters; included residents, undergraduate medical students, trainees, or any other non-MD participants in the CPD interventions; had no evaluation component; or had no CPD intervention.

### Data charting

We utilized Microsoft Excel to develop a data charting template. The data charting template included study characteristics (e.g., country, setting, medical specialty, study design andstructure, delivery mode, topic, use of theory in program development), and details of the evaluation (e.g., use of an evaluation model/framework, type of evaluation, and outcomes of the evaluation). DL and FK used this template to chart the data for all included studies. SH and LA then independently reviewed half of the charted necessary to assess author agreement and reach consensus.

### Collating, summarizing, and reporting the data

Once data charting was complete, the study characteristics were quantified using quantitative content analysis. We aimed to use a quality checklist to determine the quality of the included evaluations. However, upon exploration of the existing quality checklists, we discovered that while there are several with an educational focus, they focus on the overall study quality, as opposed to the quality of the evaluation conducted. We therefore developed a checklist to assess evaluation quality using a program evaluation lens. We used this lens to identify the gaps in current practice and highlight any particularly rigorous program evaluations. The checklist was developed by combining salient elements from the SQUIRE 2.0 Revised Standards for Quality Improvement Reporting Excellence [11], SQUIRE-EDU Standards for Quality Improvement Reporting Excellence in Education [12], and the TREND Statement checklist (for non-randomized evaluations of interventions) [13]. This was done as not all their components were relevant to evaluation quality. We chose these quality reporting tools as they are widely used. Two CPD experts reviewed the checklist and provided feedback, which was used to refine the checklist, but no items were added or removed.

The final checklist included 13 items, and for each item reviewers assigned it either a yes (met the criteria for the category), no (did not meet the criteria for the category), or partially (somewhat met the criteria for the item). These items were arranged across six categories of CPD intervention, methods (evaluation methodology, measures, analysis), results, and discussion.

For each item, along with its description, the checklist included the rationale for the item as well as the item source (e.g., adapted from an existing checklist, or author developed). For a detailed description of the checklist, refer to Appendix 2. SH and LA independently completed the checklist for a quarter of the studies and then discussed decisions, including differences. Through this process additional notes were added to the checklist to help guide decision making with the aim of better aligning their completion of the checklist. The remaining studies were split evenly between SH and LA. Any uncertainties were resolved through discussion until consensus was reached. As this quality checklist aimed to explore what is being done well in program evaluation, and what needed improvement, rather than produce a score for each study, we narratively summarized the proportion of answers for each category.

### Expert consultation

After initial analysis, expert reviewers reviewed the draft findings. The experts were key researchers in CPD and HPE. Seven experts reviewed an audio-recorded PowerPoint of this study’s rationale, methods, results, and discussion. The experts completed a 5-question Google form survey that inquired if the results aligned with what they would have expected, how to best present the findings, what to focus on in the discussion, and their thoughts on the most relevant elements of the findings to practice.

## Results

**Figure 1:**
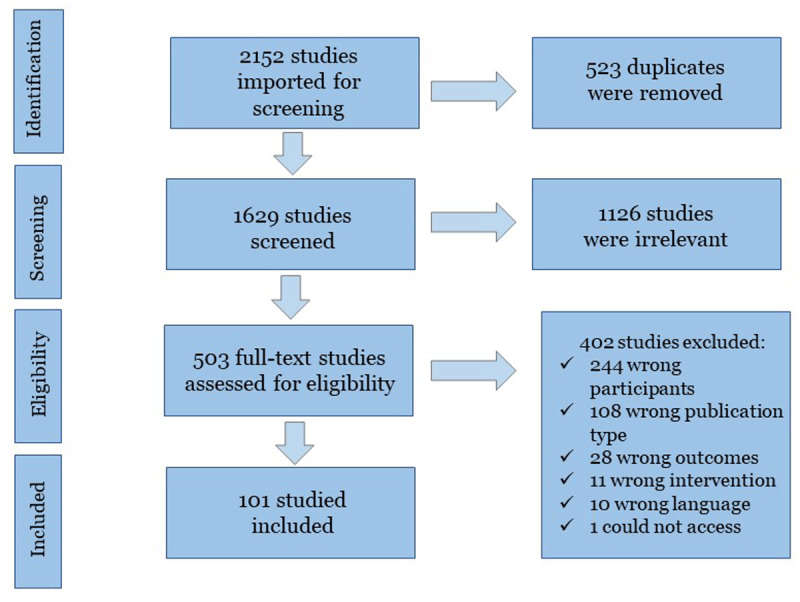
Flowchart of the literature search and study selection process.

The searches returned 2152 articles; 101 studies were included (See Figure 1). More than half of the studies originated from North America, were in hospital, or community settings, and many had general practitioners as their primary participants (See Table 1). Studies were published across the entire study period with the most appearing in 2020 (n = 13).

**Table 1:** Characteristics of included studies.


STUDY CHARACTERISTIC	NUMBER OF STUDIES (%)

*Country of Study*	

USA	42 (41.6)

Canada	9 (8.9)

UK	8 (7.9)

Australia	5 (5.0)

Japan	4 (4.0)

China, Germany, Italy, Iran, Netherlands	3 (3.0)

Brazil, France, India, International	2 (2.0)

Ireland, Israel, Norway, Qatar, Saudi Arabia, South Africa, Spain, Sweden, Taiwan, Vietnam	1 (1.0)

*Setting*	

Hospital	47 (46.5)

Community	27 (26.7)

University	14 (13.9)

Other (nationwide, professional associations, private Organizations, Military, Not Clear)	11 (8.4)

*Specialty*	

GP/Primary Care Physician	38 (37.6)

Surgeon	16 (15.8)

Varied Specialties	14 (13.9)

Physician – not specified	7 (6.9)

Pediatrician	6 (5.9)

Internist	4 (4.0)

Hospitalist	3 (3.0)

Anesthesiologist, Oncologist, Psychiatrist, Radiologist	2 (2.0)

Cardiologist, Emergency Medicine, ICU, Pathologist, Rheumatologist	1 (1.0)


*Total percent greater than 100 as multiple options could exist per program.

The included studies included a variety of study designs with pre-post test designs (n = 68; 67.3%) most represented followed by post-test only (n = 25; 24.8%). See Table 2 for study design characteristics.

**Table 2:** Design characteristics of included studies.


STUDY DESIGN CHARACTERISTIC	NUMBER OF STUDIES (%)

*Study Design*	

Pre-post test	68 (67.3)

Post-test only	25 (24.8)

Randomized Controlled Trial	4 (4.0)

Retrospective	2 (2.0)

Ethnographic, Prospective	1 (1.0)

*Method*	

Quantitative	58 (57.4)

Mixed methods	30 (29.7)

Qualitative	13 (12.9)

Type of Measure*	

Unvalidated survey	58 (57.4)

Interview	25 (24.8)

Record Review	19 (18.8)

Validated survey (*reported survey validity data*)	11 (10.9)

Performance Rating	11 (10.9)

Open-ended questions	10 (9.9)

Other	8 (7.9)

Focus Groups	7 (6.9)

Multiple Choice Questions- knowledge test questions	4 (4.0)

Patient Questionnaire	2 (2.0)

*Evaluation Framework*	

None specified	91 (90.1)

Kirkpatrick	7 (6.9)

Modified Kirkpatrick	2 (2.0)

Realist	1 (1.0)


*Total percent greater than 100 as multiple options could exist per program.

The CPD programs included various foci (skill development, scholarship, teaching, leadership, mentoring, promoting well-being), types (interactive, didactic, mixed, other), modes of delivery (in class, online, hybrid), and number of session offerings (multiple, one-time) (See Table 3).

**Table 3:** Characteristics of Continuing Professional Development (CPD) programs.


CHARACTERISTIC	NUMBER OF STUDIES (%)

*Topic*	

Skill development	81 (80.2)

Scholarship	13 (12.9)

Teaching	6 (5.9)

Leadership	4 (4.0)

Mentoring	2 (2.0)

Well-being	1 (1.0)

*CPD Program Type*	

Mixed**	66 (65.3)

Interactive***	19 (18.8)

Didactic	11 (10.9)

Other	3 (3.0)

Not specified	2 (2.0)

CPD Program Delivery*	

In person	61 (60.4)

Hybrid	25 (24.8)

Online	16 (15.8)

Not reported	1 (1.0)

*CPD Program Number of Sessions*	

Multiple	60 (59.4)

One-off	29 (28.7)

Not specified	12 (11.9)


*Total percent greater than 100 as multiple options could be selected. **Mixed = combination of didactic and interactive learning. ***Interactive = learners actively involved in learning.

### Evaluation Quality

We quantitatively summarized the frequencies of studies meeting each criterion across three categories (no, partial, yes). No study received a ‘yes’ for all the 14 criteria, with only one study [15] scoring ‘yes’ for 13 of the criteria. Most studies (n = 97, 96%) received a ‘yes’ for six or less of the criteria, with approximately half only receiving a ‘yes’ for one or less of the criteria (n = 45, 45%).

A few studies met only a limited number of the included criteria for evaluation techniques. For instance, most studies (81%) either did not use theories to elaborate on their CPD intervention design (n = 39) or had only explicated their program design in the context of current evidence (n = 44) without using a theory to explain why they believed their CPD intervention would work. Those that incorporated theory, referred to the following: the adult learning theory [16], theory of planned behavior [16], behavior change model [17], implementation change model [18], dual process theory [19], script theory [19], development process theoretical framework [20], social marketing theory [21], apprenticeship model [22], and the Comskil model [14].

The majority (88%) of the studies did not use an evaluation framework and among those that did, the Kirkpatrick framework was the dominant framework. Only one study used the realist framework to investigate the intervention processes [15]. Further, 95% of evaluations did not provide a rationale for their evaluation method or justify why the selected evaluation approach would be appropriate based on their evaluation goals. The frequencies of studies meeting each criterion are illustrated in Table 4 (See Appendix 3 for Table 4).

### Expert Consultations

The consulted experts did not find the results unexpected, agreed with our presentation of results, and did not believe any key trends or literature were missing. Their input helped shape the focus of the discussion centering on how the methodological rigor of evaluations can be improved given the findings. Our experts emphasized the importance of highlighting the underutilization of theoretical underpinning and evaluation frameworks, insufficient elaboration on associations between intervention processes and outcomes, and absence of measures that would capture unintended outcomes.

## Discussion

This review sought to answer the following questions: *a) What evaluation techniques are being used to evaluate CPD programs for physicians, and how are these techniques being implemented?* and *b) What is the quality of evaluations of CPD programs for physicians?*

In terms of the evaluation techniques used, this review showed that few studies clearly articulated the frameworks that they utilized with only 10 studies clearly articulating the framework that they had used, and another 2 partially articulating this [23,24]. This is problematic since research has specifically called for the use of evaluation frameworks in program evaluation efforts [5,6,7]. For evaluation implementation techniques and regardless of whether an evaluation framework was specified or not, outcome evaluation was far more common than more holistic program evaluation. This aligns with recent research that shows evaluation in HPE is largely outcome evaluation [4], however, outcome-oriented approaches can potentially fail to provide a holistic presentation of the mechanistic processes that may have contributed to the occurrence of the outcomes or lack thereof. Outcome evaluations tend to focus on evaluation approaches with linear causal relationship assumptions about the program elements and outcomes and overlook program’s theory and the complexity of the educational interventions. In turn, such evaluations may only provide information regarding the educational interventions’ outcomes but lack the ability to inform the processes through which the desired or unintended outcomes occur and why these outcomes are or are not observed [25,26].

Research has both demonstrated and underlined the use of theory (e.g., social theory of learning) in elucidating the potential impacts associated with CPD programs [27]. In particular, the use of complexity and system theories has been encouraged due to the complex interplay of multiple interacting components as well as the non-linear relationships between the intervention elements and outcomes in HPE [25]. Educators and evaluators need to pay close attention to the theories underpinning evaluation frameworks and whether they align with their specific evaluation objectives when determining which evaluation framework to employ. The use of theory-driven evaluations, such as the realist framework, is encouraged in conjunction with more outcome-focused evaluation models since theory-based evaluations allow for elucidating the mechanisms that connect program processes to its outcomes and why certain outcomes may or may not be occurring.

Implementation science researchers have recognized the need for determining interventions’ theoretical bases that would assist with understanding the mechanisms of change at play [28,29,30]. This work can help inform evaluation in HPE. For example, Nilsen (2015) introduced a framework that entailed three main aims for using theoretical approaches that are used in implementing interventions: *a) describing/guiding the process of translating research into practice; b) understanding/explaining what influences implementation outcomes;* and *c) evaluating implementation*. These three aims were further broken down to five categories of theories, models, and frameworks: *process models, determinant frameworks, classic theories, implementation theories*, and *evaluation frameworks*. The authors asserted several advantages associated with the use of formal theories in developing interventions including the ability to openly question, challenge, refine, adapt, or refute theories; a greater degree of consistency between theories and the existing facts; and the ability of theories to offer meaningful contexts to facts, promoting the construction of a unified body of knowledge [31].

In terms of the quality of CPD evaluations, we developed and utilized a quality checklist to determine the quality of the evaluations conducted. We found that when using a program evaluation lens to assess the quality of evaluations, the evaluation quality for most studies was low. This was true regardless of study type, while the mean quality checklist score was higher for qualitative studies (mean = 14), than quantitative studies (mean = 7) and mixed method studies (mean = 10), the mean scores were at most just over half of the maximum score. This was unsurprising, as quantitative studies often focus on outcomes only, while half of the mixed methods studies used less in-depth methods of qualitative data collection such as open-ended questions and record review.

Assessing the studies against the checklist allowed us to depict a better presentation of the current state of the literature, highlight gaps, underline exemplar CPD evaluation approaches, and offer future recommendations. Prominent gaps pertaining to the conduct and reporting of CPD evaluations included: inadequate attention paid to theory in intervention design; unclear descriptions of evaluation models; emphasis on the categorization of program outcome evaluation and minimal utilization of evaluation models that include more than simple classification of outcomes; infrequent reporting of rationale for choosing specific methods for analysis; limited reporting of unintended consequences and links between the interventions and contextual factors; as well as insufficient attention paid to the interventions’ causal pathways and interpretation of the findings in the context of current evidence and theory. Table 5 offers some considerations on how to address these gaps with related rationale. Table 5 also illustrates an example using an exemplar study that received the highest score from our checklist, to illustrate how these considerations can be done in practice.

**Table 5:** Considerations on how to improve evaluation in continuing professional development (CPD).


CONSIDERATION 1 – CONSIDER WHY YOU HAVE CHOSEN A PARTICULAR EVALUATION MODEL AND BE EXPLICIT WITH YOUR RATIONALE.

Importance: Justifying the use of a particular evaluation model allows evaluators to examine if the model is best placed to help understand how and why outcomes are occurring. Communicating this in published evaluations gives transparency to the evaluation methods and allows readers to gain insight into why a model was chosen, helping them to make a judgment on the appropriateness of the evaluation.

*Example:* McDaniel and colleagues utilize realist evaluation to evaluate a clinical faculty mentorship program. Realist evaluation is one of many types of program evaluation that allows for exploration of outcomes and helps to explain how and why they occur. They explain what realist evaluation is, why they took a qualitative approach as a result of using realist evaluation and the three-phase realist evaluation process they employed. They also explain the underlying concept of realist evaluation which focuses on context, mechanisms, and outcomes. Their full explanation can be found on page 105 under the conceptual framework section of the methods [15].

CONSIDERATION 2 – BE CLEAR ON WHY THE CPD PROGRAM IS EXPECTED TO WORK (E.G., PROGRAM THEORY).

Importance: Program theory is central to evaluation. Developing program theory should be part of the development of educational intervention. It is often developed and refined in an iterative process. Developing and communicating program theory allows evaluators (and readers of published evaluations) to understand how and why the education intervention is expected to work. Not only does it provide a foundation for conducting the evaluation, but also helps those wishing to implement a similar intervention understand how the program is leading to the outcomes.

*Example –* McDaniel and colleagues present a program theory that draws on contexts, mechanisms, and outcomes. This is in line with their use of a realist evaluation model, as mentioned in consideration 1. While the program theory is iterative, articulating the initial program theory guides both the development of the educational intervention and the evaluation. It also provides the readers insight into why and how the authors thought the educational intervention would work. The program theory is on page 105–106 [15].

CONSIDERATION 3 – MEASURE PROCESSES AS WELL AS OUTCOMES, TAKING A BROAD VIEW OF OUTCOMES CONSIDERING UNINTENDED OUTCOMES AND CONTEXTUAL AND EXTERNAL FACTORS TO HELP UNDERSTAND HOW AND WHY OUTCOMES ARE OCCURRING.

Importance: Capturing information about the development and implementation of the intervention (processes) is crucial to understanding how and why the program outcomes did or did not occur, as well as why any unintended outcomes might have resulted. This information allows us to understand if the intervention was implemented as intended and what else was happening that may have contributed to the outcomes, or that might be a reason for why outcomes were not observed. Additionally, educational interventions do not occur in a bubble, therefore contextual factors such as resources, culture, and level of support (just to name a few) can affect whether outcomes do or do not occur, and as such it is important to take these into consideration. The collection of this information can be informed by program theory and program evaluation models.

*Example:* The McDaniel and colleagues’ paper would have benefited from exploration of the processes of the intervention – that it was the mentorship program implemented as intended – however they do describe multiple barriers to the program which speaks to implementation, and why some may not have experienced particular outcomes (page 110). They do a good job of capturing both unintended outcomes, as well as contextual and external factors that help understand how the outcomes occurred. This is because realist evaluation explicitly calls out the importance of context as a key element of the program theory. They identified four context domains: (1) past personal experience, (2) current competing priorities, (3) institutional culture, and (4) gaps in support and resources that influenced the outcomes. They also identified three outcomes that were not the same as those articulated in the initial program theory. For a full description of the contexts and outcomes see the results section of the paper, pages 106–110 [15].

CONSIDERATION 4 – LINK RESULTS BACK TO PROGRAM THEORY AND DESCRIBE MECHANISMS FOR HOW AND WHY INTERVENTIONS DID OR DID NOT WORK.

Importance: The results should be linked back to the program theory, and consideration should be given to whether the initial program theory holds, or whether revision of the theory is required. Results should be discussed in the context of the program theory, and they should elaborate on potential reasons as to why certain outcomes did or did not occur. This allows consideration of if it was the intervention leading to the outcomes, or if there were other factors at play.

*Example:* McDaniel and colleagues address the mechanisms for how and why the program worked, or didn’t work in the mechanisms, barriers, and revised program theory sections of the results. The mechanisms include connecting with faculty, sharing ideas and strategies and self-reflecting. And the barriers include time and location limitations, perceived lack of fit, and individual priorities. While the revised program theory focuses on positive outcomes, the presentation of the barriers helps to understand why some participants of the program may not have experienced these outcomes. For full details see pages 108–110 [15].


This review revealed considerable opportunities for improving the quality of CPD evaluations. One reason for less attention paid to these elements when conducting CPD evaluations might be that it is only recently HPE journals are recognizing and requiring inclusion of theory in the submissions they publish. Furthermore, recognition of contextual elements in evaluations is another recent phenomenon particularly in CPD where few studies have used approaches that help understand how and why impacts may be occurring rather than merely ‘did impacts occur?’ Moreover, compared to Undergraduate Medical Education (UME), a great number of CPD offices tend to have less access to doctoral-level trained educators and many working in this space may not have the required training to undertake more rigorous evaluations. We also imagine that there may be a lag in the uptake of key reporting guidelines like the SQUIRE-EDU extension that could help improve outcomes reporting when evaluating CPD.

Although access to limited resources and evaluation expertise may contribute to the lack of evaluation framework use, and the use of simple taxonomies of program outcomes when frameworks are used, it is important to note that the short-term effects such as increased knowledge do not necessarily result in more long-term outcomes such as practice changes, enhanced care quality or patient-related outcomes [32,33]. More long-term program evaluations that pay simultaneous attention to both processes (including contextual/external elements) and impacts (including unintended outcomes) of the CPD interventions and incorporate the use of theory using program evaluation models such as the logic model, realist evaluation, CIPP, contribution analysis, layered analysis, among several others, are needed.

Moreover, in line with enhancing the quality of CPD evaluations, evaluation utility is an important point worth emphasizing. Keeping evaluation use at the forefront of the intervention design, development, and evaluation from program conception and design to evaluation will contribute to the evaluation quality and relevance for use by stakeholders. If users are to consider the evaluation findings, evaluation utility would need to accompany every step of the evaluation process [34,35]. Lastly, articulation of causal mechanisms and processes elucidating how and why programs work or not is critical and needs to be undertaken using theory-driven evaluations [36,37,38]. CPD programs are a significant component of the complex HPE pedagogical interventions that if evaluated properly, would allow for further reform and positive change. In doing so, undertaking rigorous evaluations with strong theoretical underpinning and use of evaluation frameworks that pay close attention to these contextual and mechanistic complexities is crucial.

### Limitations

The present review included focusing on articles published after 2010. Nevertheless, this cut-off was selected to be current with the topical trends of CPD program evaluation. Further, this review may have overlooked those studies that did not specify their interventions as CPD, or those not published in English. Finally, although it would be interesting to see a comparison between MDs and non-MDs, this went beyond the scope of our review, which was originally conceived to include MDs only.

## Conclusion

This scoping review examined the span and quality of CPD evaluation techniques, and their methods of implementation in CPD programs for physicians. We developed a qualitative checklist using existing prominent checklists, that could further enhance the quality of future CPD program evaluations, which may guide studies that examine CPD program development and evaluation and highlight existing gaps associated with the evaluation of CPD programs. Salient areas in need of deliberation highlight considerations of broader and more long-term impacts of the interventions and their processes, use of theory and evidence when designing and evaluating the CPD programs, adherence to evaluation frameworks, augmenting quantitative approaches with qualitative measures to detect unintended outcomes, and lastly reflection on the potential causal pathways through which the programs may exert their impacts.

## Additional Files

The additional files for this article can be found as follows:

10.5334/pme.838.s1 Supplemental File 1. List of included studies.

10.5334/pme.838.s2 Appendix 1. PubMed Search.

10.5334/pme.838.s3 Appendix 2. Qualitative Checklist.

10.5334/pme.838.s4 Appendix 3. Table 4. Checklist for quality appraisal of Continuing Professional Development (CPD) program evaluations in individual studies.
