# Supplementary material for: Evaluation of Continuing Professional Development for Physicians – Time for Change: A Scoping Review
Source: Perspect Med Educ. 2023 Jun 2;12(1):198–207. doi: 10.5334/pme.838 (PMC10237247; doi:10.5334/pme.838)
Supplement: Supplemental File 1. — List of included studies. [file pme-12-1-838-s1.pdf]

Supplementary File 1 (List of included studies)

1. Abdullah I, Jafta AD, Chapanduka ZC. The impact of physician education regarding the importance of providing complete clinical information on the request forms of thrombophilia-screen tests at Tygerberg hospital in South Africa. *PLoS One*. 2020;15(8):e0235826. doi:10.1371/journal.pone.0235826

2. Adams RE, Laraque D, Chemtob CM, Jensen PS, Boscarino JA. Does a one-day educational training session influence primary care pediatricians' mental health practice procedures in response to a community disaster? Results from the reaching children initiative (RCI). *Int J Emerg Ment Health*. 2013;15(1):3-14.

3. Aghamirsalim M, Mehrpour SR, Kamrani RS, Sorbi R. Effectiveness of educational intervention on undermanagement of osteoporosis in fragility fractures. *Archives of Orthopaedic and Trauma Surgery*. 2012;132(10):1461-1465. doi:10.1007/s00402-012-1569-5

4. Ahmed M, Arora S, Baker P, Hayden J, Vincent C, Sevdalis N. Building capacity and capability for patient safety education: a train-the-trainers programme for senior doctors. *BMJ Qual Saf*. 2013;22(8):618-625. doi:10.1136/bmjqs-2012-001626

5. Alshammary SA, Ratnapalan S, Akturk Z. Continuing medical education as a national strategy to improve access to primary care in Saudi Arabia. *J Educ Eval Health Prof*. 2013;10:7. doi:10.3352/jeehp.2013.10.7

6. Anders MP, Fengler S, Volkmer B, Greinert R, Breitbart EW. Nationwide skin cancer screening in Germany: Evaluation of the training program. *Int J Dermatol*. 2017;56(10):1046-1051. doi:10.1111/ijd.13688

7. Baldacci S, Maio S, Simoni M, et al. The ARGA study with general practitioners: impact of medical education on asthma/rhinitis management. *Respir Med*. 2012;106(6):777-785. doi:10.1016/j.rmed.2012.02.013

8. Balmer DF, Richards BF. Faculty Development as Transformation: Lessons Learned From a Process-Oriented Program. *Teaching and Learning in Medicine*. 2012;24(3):242-247. doi:10.1080/10401334.2012.692275

9. Barnabe C, Kherani RB, Appleton T, Umaefulam V, Henderson R, Crowshoe L. Participant-reported effect of an Indigenous health continuing professional development initiative for specialists. *BMC Med Educ*. 2021;21(1):116. doi:10.1186/s12909-021-02551-9

10. Beckman HB, Wendland M, Mooney C, et al. The impact of a program in mindful communication on primary care physicians. *Acad Med*. 2012;87(6):815-819. doi:10.1097/ACM.0b013e318253d3b2

11. Bird GC, Marian K, Bagley B. Effect of a performance improvement CME activity on management of patients with diabetes. *J Contin Educ Health Prof*. 2013;33(3):155-163. doi:10.1002/chp.21180

12. Boddi M, Barbani F, Abbate R, et al. Reduction in deep vein thrombosis incidence in intensive care after a clinician education program. *J Thromb Haemost*. 2010;8(1):121-128. doi:10.1111/j.1538-7836.2009.03664.x

13. Bonabi M, Mohebbi SZ, Martinez-Mier EA, Thyvalikakath TP, Khami MR. Effectiveness of smart phone application use as continuing medical education method in pediatric oral health care: a randomized trial. *BMC Med Educ*. 2019;19(1):431. doi:10.1186/s12909-019-1852-z

14. Bylund CL, Alyafei K, Afana A, et al. Satisfaction with a 2-day communication skills course culturally tailored for medical specialists in Qatar. *J Family Community Med*. 2017;24(2):122-127. doi:10.4103/2230-8229.205118

15. Carey JN, Caldwell AM, Coughlin RR, Hansen S. Building Orthopaedic Trauma Capacity: IGOT International SMART Course. *J Orthop Trauma*. 2015;29 Suppl 10:S17-9. doi:10.1097/bot.0000000000000412

16. Carlson K, Ashford A, Hegagi M, Vokoun C. Peer Coaching as a Faculty Development Tool: A Mixed Methods Evaluation. *J Grad Med Educ*. 2020;12(2):168-175. doi:10.4300/jgme-d-19-00250.1

17. Carney PA, Abraham L, Cook A, et al. Impact of an educational intervention designed to reduce unnecessary recall during screening mammography. *Acad Radiol*. 2012;19(9):1114-1120. doi:10.1016/j.acra.2012.05.003

18. Carroll JC, Grad R, Allanson JE, et al. The Gene Messenger Impact Project: An Innovative Genetics Continuing Education Strategy for Primary Care Providers. *J Contin Educ Health Prof*. 2016;36(3):178-185. doi:10.1097/ceh.0000000000000079

19. Casey AN, Islam MM, Schütze H, et al. GP awareness, practice, knowledge and confidence: evaluation of the first nation-wide dementia-focused continuing medical education program in Australia. *BMC Fam Pract*. 2020;21(1):104. doi:10.1186/s12875-020-01178-x

20. Castel OC, Ezra V, Alperin M, et al. Can outcome-based continuing medical education improve performance of immigrant physicians? *J Contin Educ Health Prof*. 2011;31(1):34-42. doi:10.1002/chp.20099

21. Chinnaiyan KM, Peyser P, Goraya T, et al. Impact of a continuous quality improvement initiative on appropriate use of coronary computed tomography angiography. Results from a multicenter, statewide registry, the Advanced Cardiovascular Imaging Consortium. *J Am Coll Cardiol*. 2012;60(13):1185-1191. doi:10.1016/j.jacc.2012.06.008

22. Chou DTS, Solomon LB, Costi K, Pannach S, Holubowycz OT, Howie DW. Structured-mentorship Program for Periacetabular Osteotomy Resulted in Few Complications for a Low-volume Pelvic Surgeon. *Clin Orthop Relat Res*. 2019;477(5):1126-1134. doi:10.1097/corr.0000000000000571

23. Crabtree JH, Penner T, Armstrong SW, Burkart J. Peritoneal Dialysis University for Surgeons: A Peritoneal Access Training Program. *Perit Dial Int*. 2016;36(2):177-181. doi:10.3747/pdi.2015.00013

24. Cunningham DE, Zlotos L. Ten years of practice-based small group learning (PBSGL) in Scotland - a survey of general practitioners. *Educ Prim Care*. 2016;27(4):306-313. doi:10.1080/14739879.2016.1179597

25. Deed G, Kilov G, Phillips P, et al. Peer-to-Peer, Interactive GP Education can Reduce Barriers to Best Practice in Diabetes Management. *Diabetes Ther*. 2016;7(1):153-161. doi:10.1007/s13300-016-0156-0

26. Del Poggio P, Olmi S, Ciccarese F, et al. A training program for primary care physicians improves the effectiveness of ultrasound surveillance of hepatocellular carcinoma. *Eur J Gastroenterol Hepatol*. 2015;27(9):1103-1108. doi:10.1097/meg.0000000000000404

27. Dennis E, Banks P, Murata LB, et al. Validation of an electronic program for pathologist training in the interpretation of a complex companion diagnostic immunohistochemical assay. *Hum Pathol*. 2016;56:194-203. doi:10.1016/j.humpath.2016.06.013

28. Donovan AK, Wood GJ, Rubio DM, Day HD, Spagnoletti CL. Faculty Communication Knowledge, Attitudes, and Skills Around Chronic Non-Malignant Pain Improve with Online Training. *Pain Med*. 2016;17(11):1985-1992. doi:10.1093/pm/pnw029

29. Dort J, Trickey A, Paige J, Schwarz E, Dunkin B. Hands-on 2.0: improving transfer of training via the Society of American Gastrointestinal and Endoscopic Surgeons (SAGES) Acquisition of Data for Outcomes and Procedure Transfer (ADOPT) program. *Surg Endosc*. 2017;31(8):3326-3332. doi:10.1007/s00464-016-5366-z

30. Dort J, Trickey A, Paige J, et al. All in: expansion of the acquisition of data for outcomes and procedure transfer (ADOPT) program to an entire SAGES annual meeting hands-on hernia course. *Surg Endosc*. 2018;32(11):4491-4497. doi:10.1007/s00464-018-6196-y

31. Douglass K, Williams A. Faculty Development Program for Emergency Medicine Physicians in India: A Pilot Program. *AEM Educ Train*. 2019;3(1):33-38. doi:10.1002/aet2.10125

32. Dudek NL, Marks MB, Bandiera G, White J, Wood TJ. Quality in-training evaluation reports--does feedback drive faculty performance? *Acad Med*. 2013;88(8):1129-1134. doi:10.1097/ACM.0b013e318299394c

33. Duke SL, Tan LT, Jensen NBK, et al. Implementing an online radiotherapy quality assurance programme with supporting continuous medical education - report from the

EMBRACE-II evaluation of cervix cancer IMRT contouring. *Radiother Oncol*. 2020;147:22-29. doi:10.1016/j.radonc.2020.02.017

34. Dyrkorn R, Langaas HC, Giverhaug T, Espnes KA, Rowett D, Spigset O. Academic detailing as a method of continuing medical education. *Adv Med Educ Pract*. 2019;10:717-725. doi:10.2147/amep.S206073

35. Fassiotto M, Maldonado Y, Hopkins J. A long-term follow-up of a physician leadership program. *J Health Organ Manag*. 2018;32(1):56-68. doi:10.1108/jhom-08-2017-0208

36. Fheodoroff K, Bhidayasiri R, Jacinto LJ, et al. Ixcellence Network®: an international educational network to improve current practice in the management of cervical dystonia or spastic paresis by botulinum toxin injection. *Funct Neurol*. 2017;32(2):103-110. doi:10.11138/fneur/2017.32.2.103

37. Fleming GM, Simmons JH, Xu M, et al. A Facilitated Peer Mentoring Program for Junior Faculty to Promote Professional Development and Peer Networking. *Acad Med*. 2015;90(6):819-826. doi:10.1097/acm.0000000000000705

38. Foley T, Jennings A, Boyle S, Smithson WH. The development and evaluation of peer-facilitated dementia workshops in general practice. *Educ Prim Care*. 2018;29(1):27-34. doi:10.1080/14739879.2017.1390693

39. Foster JD, Gash KJ, Carter FJ, et al. Development and evaluation of a cadaveric training curriculum for low rectal cancer surgery in the English LOREC National Development Programme. *Colorectal Dis*. 2014;16(9):O308-19. doi:10.1111/codi.12576

40. Fujimori M, Shirai Y, Asai M, et al. Development and preliminary evaluation of communication skills training program for oncologists based on patient preferences for communicating bad news. *Palliat Support Care*. 2014;12(5):379-386. doi:10.1017/s147895151300031x

41. Garg K, Manjunatha N, Kumar CN, Chand PK, Math SB. Case vignette-based evaluation of psychiatric blended training program of primary care doctors. *Indian J Psychiatry*. 2019;61(2):204-207. doi:10.4103/psychiatry.IndianJPsychiatry_250_18

42. Golestaneh L, Neugarten J, Southern W, Kargoli F, Raff A. Improving the diagnostic workup of hyponatremia in the setting of kidney disease: a continuing medical education (CME) initiative. *Int Urol Nephrol*. 2017;49(3):491-497. doi:10.1007/s11255-017-1501-6

43. Gorsche RG, Woloschuk W. Rural physicians’ skills enrichment program: a cohort control study of retention in Alberta. *Aust J Rural Health*. 2012;20(5):254-258. doi:10.1111/j.1440-1584.2012.01298.x

44. Greenberg CC, Ghousseini HN, Pavuluri Quamme SR, et al. A Statewide Surgical Coaching Program Provides Opportunity for Continuous Professional Development. *Ann Surg*. 2018;267(5):868-873. doi:10.1097/sla.0000000000002341

45. Gu J, Zhu SZ, Chen TJ, et al. Evaluation of the Spring Seedling Project-Zhaotong Program: A study of a novel continuing medical education program for rural doctors in China. *Aust J Rural Health*. 2020;28(5):434-442. doi:10.1111/ajr.12659

46. Gugenheim J, Debs T, Gravié JF, et al. Results of the FUSE Evaluation Project in France. *Surg Endosc*. 2020;34(4):1819-1822. doi:10.1007/s00464-019-06938-0

47. Guo FR, Hung LY, Chang CJ, Leung KK, Chen CY. The evaluation of a Taiwanese training program in smoking cessation and the trainees' adherence to a practice guideline. *BMC Public Health*. 2010;10:77. doi:10.1186/1471-2458-10-77

48. Halverson AL, DaRosa DA, Borgstrom DC, et al. Evaluation of a blended learning surgical skills course for rural surgeons. *Am J Surg*. 2014;208(1):136-142. doi:10.1016/j.amjsurg.2013.12.039

49. Houwink EJ, Muijtjens AM, van Teeffelen SR, et al. Effect of comprehensive oncogenetics training interventions for general practitioners, evaluated at mulconsideration le performance levels. *PLoS One*. 2015;10(4):e0122648. doi:10.1371/journal.pone.0122648

50. Ivanovic J, Anstee C, Ramsay T, et al. Using surgeon-specific outcome reports and positive deviance for continuous quality improvement. *Annals of Thoracic Surgery*. 2015;100(4):1188-1195. doi:10.1016/j.athoracsur.2015.04.012

51. Job Jr PM, Von Bahten LC, De Oliveira Jr N. Evaluation of the effectiveness of systematized training of advanced trauma life support protocol in the interpretation of cervical spine and chest radiographs in three different emergency services. *Journal of Trauma - Injury, Infection and Critical Care*. 2011;70(6):E122-E124. doi:10.1097/TA.0b013e3181bbd721

52. Joyner J, Moore MA, Simmons DR, et al. Impact of performance improvement continuing medical education on cardiometabolic risk factor control: the COSEHC initiative. *J Contin Educ Health Prof*. 2014;34(1):25-36. doi:10.1002/chp.21217

53. Kadlec H, Hollander MJ, Clelland C, Kallstrom L, Hollander M. Family physicians enhance end-of-life care: evaluation of a new continuing medical education learning module in British Columbia. *BMC Med Educ*. 2015;15:119. doi:10.1186/s12909-015-0392-4

54. Kang H, Cui ZS, Chia J, et al. Innovative Multimodal Training Program for Family Physicians Leads to Positive Outcomes Among Their HIV-Positive Patients. *J Contin Educ Health Prof*. 2018;38(1):60-65. doi:10.1097/ceh.0000000000000189

55. Kerr AM, Kachmar U, Palocko B, Biechler M, Shaub T. "Confessions of a Reluctant Caregiver" Palliative Educational Program: The Results of a Survey Assessing Physicians' Perceptions of Drama-Based Education for End-of-Life Care. *J Cancer Educ*.:4. doi:10.1007/s13187-019-01655-7

56. Koffarnus RL, Mican LM, Lopez DA, Barner JC. Evaluation of an inpatient psychiatric hospital physician education program and adherence to American Diabetes Association

practice recommendations. *Am J Health Syst Pharm*. 2016;73(5 Suppl 1):S57-62. doi:10.2146/sp150037

57. Kok R, Hoving JL, Smits PB, Ketelaar SM, van Dijk FJ, Verbeek JH. A clinically integrated post-graduate training programme in evidence-based medicine versus “no intervention” for improving disability evaluations: a cluster randomised clinical trial. *PLoS One*. 2013;8(3):e57256. doi:10.1371/journal.pone.0057256

58. Konishi E, Saiki T, Kamiyama H, et al. Improved cognitive apprenticeship clinical teaching after a faculty development program. *Pediatr Int*. 2020;62(5):542-548. doi:10.1111/ped.14095

59. Larson MJ, Browne C, Nikitin RV, et al. Physicians report adopting safer opioid prescribing behaviors after academic detailing intervention. *Subst Abus*. 2018;39(2):218-224. doi:10.1080/08897077.2018.1449175

60. Lee BC, Ruiz-Cordell KD, Haimowitz SM, Williams C, Stambler BS, Mandarakas A. Personalized, assessment-based, and tiered medical education curriculum integrating treatment guidelines for atrial fibrillation. *Clinical Cardiology*. 2017;40(7):455-460. doi:10.1002/clc.22676

61. Lee WW, Alkureishi ML, Isaacson JH, et al. Impact of a brief faculty training to improve patient-centered communication while using electronic health records. *Patient Educ Couns*. 2018;101(12):2156-2161. doi:10.1016/j.pec.2018.06.020

62. Lynch M, McFetridge N. Practice leaders programme: entrusting and enabling general practitioners to lead change to improve patient experience. *Perm J*. 2011;15(1):28-34. doi:10.7812/tpp/10-095

63. McDaniel CE, Rooholamini SN, Desai AD, Reddy S, Marshall SG. A Qualitative Evaluation of a Clinical Faculty Mentorship Program Using a Realist Evaluation Approach. *Acad Pediatr*. 2020;20(1):104-112. doi:10.1016/j.acap.2019.08.008

64. McKinney CM, Mookherjee S, Fihn SD, Gallagher TH. An Academic Research Coach: An Innovative Approach to Increasing Scholarly Productivity in Medicine. *J Hosp Med*. 2019;14(8):457-461. doi:10.12788/jhm.3194

65. Moattari M, Yadgari D, Hoseini SJ. The evaluation of a composed program of continuing medical education for general practitioners. *J Adv Med Educ Prof*. 2014;2(3):120-125.

66. Morbach C, Buck T, Rost C, et al. Point-of-care B-type natriuretic peptide and portable echocardiography for assessment of patients with suspected heart failure in primary care: rationale and design of the three-part Handheld-BNP program and results of the training study. *Clin Res Cardiol*. 2018;107(2):95-107. doi:10.1007/s00392-017-1181-3

67. Morris L, Gorayski P, Turner S. Targeting general practitioners: Prospective outcomes of a national education program in radiation oncology. *J Med Imaging Radiat Oncol*. 2018;62(2):270-275. doi:10.1111/1754-9485.12685

68. Muller E, Diesing A, Rosahl A, Scholl I, Harter M, Buchholz A. Evaluation of a shared decision-making communication skills training for physicians treating patients with asthma: a mixed methods study using simulated patients. *BMC Health Serv Res*. 2019;19(1):10. doi:10.1186/s12913-019-4445-y

69. Pelayo M, Cebrián D, Areosa A, Agra Y, Izquierdo JV, Buendía F. Effects of online palliative care training on knowledge, attitude and satisfaction of primary care physicians. *BMC Fam Pract*. 2011;12:37. doi:10.1186/1471-2296-12-37

70. Peterson LE, Blackburn B, Phillips RL, Puffer JC. Improving quality of care for diabetes through a maintenance of certification activity: family physicians' use of the chronic care model. *J Contin Educ Health Prof*. 2014;34(1):47-55. doi:10.1002/chp.21216

71. Pimenta HB, Caldeira AP, Mamede S. Effects of 2 educational interventions on the management of hypertensive patients in primary health care. *J Contin Educ Health Prof*. 2014;34(4):243-251. doi:10.1002/chp.21252

72. Pluye P, Grad R, Granikov V, et al. Feasibility of a knowledge translation CME program: Courriels Cochrane. *J Contin Educ Health Prof*. 2012;32(2):134-141. doi:10.1002/chp.21136

73. Porcheret M, Main C, Croft P, Dziedzic K. Enhancing delivery of osteoarthritis care in the general practice consultation: evaluation of a behaviour change intervention. *BMC Fam Pract*. 2018;19(1):26. doi:10.1186/s12875-018-0715-8

74. Pradarelli JC, Jaffe GA, Lemak CH, Mulholland MW, Dimick JB. A leadership development program for surgeons: First-year participant evaluation. *Surgery*. 2016;160(2):255-263. doi:10.1016/j.surg.2016.03.011

75. Pugh CM, Arafat FO, Kwan C, et al. Development and evaluation of a simulation-based continuing medical education course: Beyond lectures and credit hours. *American Journal of Surgery*. 2015;210(4):603-609. doi:10.1016/j.amjsurg.2015.05.034

76. Rashid MA, Nicholson JG, Fazal F, et al. "Turning Point": Evaluating the Impact of a Three-Month UK-Based Clinical Education Training Programme for Physicians from a Chinese Medical School. *Adv Med Educ Pract*. 2020;11:601-607. doi:10.2147/amep.S257384

77. Reisner A, Burns TG, Hall LB, et al. Quality Improvement in Concussion Care: Influence of Guideline-Based Education. *J Pediatr*. 2017;184:26-31. doi:10.1016/j.jpeds.2017.01.045

78. Reynolds N, Wuyts P, Badger S, Fusar-Poli P, McGuire P, Valmaggia L. The impact of delivering GP training on the clinical high risk and first-episode psychosis on referrals and pathways to care. *Early Interv Psychiatry*. 2015;9(6):459-466. doi:10.1111/eip.12126

79. Saiki T, Imafuku R, Pickering J, Suzuki Y, Steinert Y. On-site Observational Learning in Faculty Development: Impact of an International Program on Clinical Teaching in

Medicine. *J Contin Educ Health Prof*. 2019;39(2):144-151. doi:10.1097/ceh.0000000000000253

80. Schaye V, Janjigian M, Hauck K, et al. A workshop to train medicine faculty to teach clinical reasoning. *Diagnosis (Berl)*. 2019;6(2):109-113. doi:10.1515/dx-2018-0059

81. Sehgal NL, Wachter RM, Vidyarthi AR. Bringing continuing medical education to the bedside: the University of California, San Francisco Hospitalist Mini-College. *J Hosp Med*. 2014;9(2):129-134. doi:10.1002/jhm.2111

82. Servoss J, Chang C, Olson D, Ward KR, Mulholland MW, Cohen MS. The Surgery Innovation and Entrepreneurship Development Program (SIEDP): An Experiential Learning Program for Surgery Faculty to Ideate and Implement Innovations in Health care. *J Surg Educ*. 2018;75(4):935-941. doi:10.1016/j.jsurg.2017.09.017

83. Shah NH, Anspacher M, Davis A, Bhansali P. Development of a Curriculum on the Child With Medical Complexity: Filling a Gap When Few Practice Guidelines Exist. *J Contin Educ Health Prof*. 2015;35(4):278-283. doi:10.1097/ceh.0000000000000001

84. Shah P, Cross V, Sii F. Sailing a safe ship: improving patient safety by enhancing the leadership skills of new consultant specialist surgeons. *J Contin Educ Health Prof*. 2013;33(3):190-200. doi:10.1002/chp.21184

85. Shah S, Toelle BG, Sawyer SM, et al. Feasibility study of a communication and education asthma intervention for general practitioners in Australia. *Aust J Prim Health*. 2010;16(1):75-80. doi:10.1071/py09056

86. Sharpe RE Jr, Huffman RI, Congdon RG, et al. Implementation of a Peer Learning Program Replacing Score-Based Peer Review in a Multispecialty Integrated Practice. *AJR Am J Roentgenol*. 2018;211(5):949-956. doi:10.2214/ajr.18.19891

87. Slekovec C, Leroy J, Vernaz-Hegi N, et al. Impact of a region wide antimicrobial stewardship guideline on urinary tract infection prescription patterns. *Int J Clin Pharm*. 2012;34(2):325-329. doi:10.1007/s11096-012-9606-6

88. Steadman RH, Burden AR, Huang YM, Gaba DM, Cooper JB. Practice Improvements Based on Participation in Simulation for the Maintenance of Certification in Anesthesiology Program. *Anesthesiology*. 2015;122(5):1154-1169. doi:10.1097/aln.0000000000000613

89. Stille CJ, Savageau JA, McBride J, Alper EJ. Quality Improvement “201”: Context-Relevant Quality Improvement Leadership Training for the Busy Clinician-Educator. *American Journal of Medical Quality*. 2012;27(2):98-105. doi:10.1177/1062860611414404

90. Swiggart WH, Bills JL, Penberthy JK, Dewey CM, Worley LLM. A Professional Development Course Improves Unprofessional Physician Behavior. *Joint Commission Journal on Quality and Patient Safety*. 2020;46(2):64-71. doi:10.1016/j.jcjq.2019.11.004

91. Takaesu Y, Watanabe K, Numata S, et al. Improvement of psychiatrists' clinical knowledge of the treatment guidelines for schizophrenia and major depressive disorders using the "Effectiveness of Guidelines for Dissemination and Education in Psychiatric Treatment (EGUIDE)" project: A nationwide d. *Psychiatry Clin Neurosci*. 2019;73(10):642-648. doi:10.1111/pcn.12911

92. Thai TTN, Nguyen KT, Pham TT, Nguyen PM, Derese A. Can combined online and face-to-face continuing medical education improve the clinical knowledge and skills of family doctors in Vietnam? A cluster randomised controlled trial. *Trop Med Int Health*. 2020;25(4):388-396. doi:10.1111/tmi.13372

93. Vaillancourt S, Schultz SE, Leaver C, Stukel TA, Schull MJ. Effect of a brief emergency medicine education course on emergency department work intensity of family physicians. *Cjem*. 2013;15(1):34-41. doi:10.2310/8000.2012.120617

94. Vasudev K, Lamoure J, Beyaert M, et al. Academic detailing among psychiatrists - feasibility and acceptability. *Int J Health Care Qual Assur*. 2017;30(1):79-88. doi:10.1108/ijhcqa-04-2016-0047

95. Wang SL, Cha HH, Lin JR, et al. Impact of Physician Education and a Dedicated Inferior Vena Cava Filter Tracking System on Inferior Vena Cava Filter Use and Retrieval Rates Across a Large US Health Care Region. *J Vasc Interv Radiol*. 2016;27(5):740-748. doi:10.1016/j.jvir.2016.01.130

96. Wang W, Chen F, Kong L, Guo Y, Cheng J, Zhang Y. Prospective Evaluation of the Accuracy of A Training Program in Image Recognition by Narrow-Band Imaging Guided Hysteroscopy of Endometrial Neoplasms. *Gynecol Obstet Invest*. 2020;85(3):284-289. doi:10.1159/000507929

97. Weber RA, Cable CT, Wehbe-Janek H. Learner Perspectives of a Surgical Educators Faculty Development Program Regarding Value and Effectiveness: A Qualitative Study. *Plast Reconstr Surg*. 2016;137(3):1057-1061. doi:10.1097/01.prs.0000475825.09531.68

98. Wekell P, Aspegren K, Holmgren D. Devising a competency-based continuing professional development programme to meet the needs of on-call consultant paediatricians. *Acta Paediatrica, International Journal of Paediatrics*. 2014;103(3):320-330. doi:10.1111/apa.12511

99. Welsh JA, Nelson JM, Walsh S, Sealer H, Palmer W, Vos MB. Brief training in patient-centered counseling for healthy weight management increases counseling self-efficacy and goal setting among pediatric primary care providers: results of a pilot program. *Clin Pediatr (Phila)*. 2015;54(5):425-429. doi:10.1177/0009922814553432

100. Wu HH, Patel KR, Caldwell AM, Coughlin RR, Hansen SL, Carey JN. Surgical Management and Reconstruction Training (SMART) Course for International Orthopedic Surgeons. *Ann Glob Health*. 2016;82(4):652-658. doi:10.1016/j.aogh.2016.06.002

101. Zhang CY, Fang F, Peng MQ, Zhao Y, Liu RX, Jia CB. Qualitative evaluation of the general practitioner chronic non-communicable diseases training programme. *BMC Med Educ*. 2020;20(1):7. doi:10.1186/s12909-020-02226-x
