# Supplementary material for: Evaluation of Continuing Professional Development for Physicians – Time for Change: A Scoping Review
Source: Perspect Med Educ. 2023 Jun 2;12(1):198–207. doi: 10.5334/pme.838 (PMC10237247; doi:10.5334/pme.838)
Supplement: Appendix 1. — PubMed Search. [file pme-12-1-838-s2.pdf]

APPENDIX 1

PubMed Search

((("Physicians" [Mesh]) OR (physician[Title/Abstract])) OR (physicians[Title/Abstract])) OR (clinician[Title/Abstract])) OR (clinicians[Title/Abstract])) OR (allergist[Title/Abstract])) OR (allergists[Title/Abstract])) OR (anesthesiologist[Title/Abstract])) OR (anesthesiologists[Title/Abstract])) OR (cardiologist[Title/Abstract])) OR (cardiologists[Title/Abstract])) OR (Dermatologist[Title/Abstract])) OR (Dermatologists[Title/Abstract])) OR (Endocrinologist[Title/Abstract])) OR (Endocrinologists[Title/Abstract])) OR (Gastroenterologist[Title/Abstract])) OR (Gastroenterologists[Title/Abstract])) OR (General Practitioner[Title/Abstract])) OR (General Practitioners[Title/Abstract])) OR (Geriatrician[Title/Abstract])) OR (Geriatricians[Title/Abstract])) OR (Hospitalist[Title/Abstract])) OR (Hospitalists[Title/Abstract])) OR (Nephrologist[Title/Abstract])) OR (Nephrologists[Title/Abstract])) OR (Neurologist[Title/Abstract])) OR (Neurologists[Title/Abstract])) OR (Oncologist[Title/Abstract])) OR (Oncologists[Title/Abstract])) OR (Ophthalmologist[Title/Abstract])) OR (Ophthalmologists[Title/Abstract])) OR (Otolaryngologist[Title/Abstract])) OR (Otolaryngologists[Title/Abstract])) OR (Pathologist[Title/Abstract])) OR (Pathologists[Title/Abstract])) OR (Pediatrician[Title/Abstract])) OR (Pediatricians[Title/Abstract])) OR (Neonatologist[Title/Abstract])) OR (Neonatologists[Title/Abstract])) OR (Physiatrist[Title/Abstract])) OR (Physiatrists[Title/Abstract])) OR (Pulmonologist[Title/Abstract])) OR (Pulmonologists[Title/Abstract])) OR (Radiologist[Title/Abstract])) OR (Radiologists[Title/Abstract])) OR (Rheumatologist[Title/Abstract])) OR (Rheumatologists[Title/Abstract])) OR (Surgeon[Title/Abstract])) OR (Surgeons[Title/Abstract])) OR (Neurosurgeon[Title/Abstract])) OR (Neurosurgeons[Title/Abstract])) OR (Urologist[Title/Abstract])) OR (Urologists[Title/Abstract])) OR (internist[Title/Abstract])) OR (internists[Title/Abstract])) OR (gynecologist[Title/Abstract])) OR (gynecologists[Title/Abstract])) OR (obstetrician[Title/Abstract])) OR (obstetricians[Title/Abstract])) OR (psychiatrist[Title/Abstract])) OR (psychiatrists[Title/Abstract])) AND (((((("education, medical, continuing"[MeSH Terms]) OR (continuing professional development[Title/Abstract])) OR (continuing interprofessional development[Title/Abstract])) OR (continuing education[Title/Abstract])) OR (faculty development[Title/Abstract])) OR (continuing medical education[Title/Abstract])))) AND ((("program evaluation"[MeSH Terms]) OR ((evaluation[Title/Abstract]) AND (program[Title/Abstract]))) OR ((evaluation[Title/Abstract]) AND (framework[Title/Abstract])))
