# Supplementary material for: Evaluation of Continuing Professional Development for Physicians – Time for Change: A Scoping Review
Source: Perspect Med Educ. 2023 Jun 2;12(1):198–207. doi: 10.5334/pme.838 (PMC10237247; doi:10.5334/pme.838)
Supplement: Appendix 2. — Qualitative Checklist. [file pme-12-1-838-s3.pdf]

APPENDIX 2

Qualitative Checklist

Checklist Item	Source(s)	Rationale
INTRODUCTION		
1. Frameworks/models/concepts/theories used in explaining the problem, developing/designing the professional development, and why the CPD was expected to work.	Adapted from SQUIRE 2.0 and Squire EDU	As outlined in SQUIRE EDU a framework/model/concept/ theory used demonstrates why the planned intervention is expected to be effective in a particular context. The key here is to make the framework/model/concept/ theory explicit. Understanding this helps readers understand why the intervention worked or didn’t work.
METHOD		
Evaluation Method		
2. Evaluation method/model/framework clearly specified for example the Kirkpatrick Model, realist evaluation, RE-AIM framework.	Author developed	When conducting an evaluation it is important to be explicit with the evaluation model, framework or method used.
3. Rationale for the evaluation method chosen provided.	Author developed	Further to explicitly stating the evaluation framework, model or method it is important to explain the rationale for choosing the particular evaluation method and why it is appropriate.
Measures		

4. Are both processes and outcomes assessed?	Adapted from SQUIRE 2.0 and Squire EDU	Helping to understand not just the outcomes of interventions but the mechanisms of change.
5. Measures chosen for studying processes **and/or** outcomes of the intervention(s), including rationale for choosing them, their operational definitions, and their validity and reliability included.	Adapted from SQUIRE 2.0 and Squire EDU	To allow for adequate description of measures chosen, and rationale for choosing them, that is why they are appropriate.
6. The chosen measures would allow for opportunities to capture unintended outcomes.	Author developed	SQUIRE 2.0, SQUIRE EDU and Trend all refer to capturing unintended outcomes in the results, this item checks whether the measures used in the study have the ability to capture unintended outcomes. It is important to understand unintended outcomes, the equivalent in a medical trial is not measuring side effects.
7. The chosen measures would allow for contextual/external elements that may contribute to success, failure, efficiency and cost of intervention.	Adapted from SQUIRE 2.0 and TREND.	Acknowledges that contextual and external factors can influence intervention processes and outcomes.
Analysis		
8. Were adequate descriptions and justifications of the chosen analysis methods provided? Were qualitative and/or quantitative methods used to draw inferences from the data?	Adapted from SQUIRE 2.0 and SQUIRE EDU	From SQUIRE EDU “a combination of quantitative and qualitative data can often help assess the fidelity, which requires record keeping not just of results but also of the reasoning for changes based on more nuanced observations. Educational improvement is a process of social change within complex systems, and this reporting

		of how the intervention changes over time provides important contextual knowledge. Simply reporting before-and-after data about course evaluations or exam scores is not enough because readers should know exactly how and why each iteration of the intervention was executed to determine whether and how they might implement similar changes in their local context."
Results and Discussion		
9. Outcomes of the CPD included with sufficient detail.	Author developed	That the outcomes are reported appropriately based on the methods and measures chosen, that there is enough detail to interpret the results.
10. Observed associations between outcomes, interventions, and relevant contextual/external elements.	SQUIRE 2.0	Links to item 7, while that focuses on if the measures chosen could capture contextual/external elements, this focuses on if they were actually captured. Important to describe the effect, if any, of contextual/external factors on processes and outcomes.
11. Did the study report unintended consequences such as unexpected benefits, problems, failures, or costs associated with the CPD?	Adapted from SQUIRE 2.0 and TREND	Links to item 6, while that focuses on if the measures chosen could capture unintended outcomes, this focuses on if they were actually captured. There is the potential to have both positive and negative unintended impacts and understanding these is important.
12. Did the study provide any/some general interpretations of the results in the context of current evidence and current theory?	Adapted from SQUIRE 2.0, SQUIRE EDU and	Links back to item 1 in the checklist. It is important to link the results to the framework/model/theory of the intervention.

	TREND	
13. Was there any discussion of results considering the mechanism by which the intervention was intended to work (causal pathways) or alternative mechanisms or explanations? (why the intervention worked or not)	SQUIRE 2.0 and TREND	Goes beyond just referring back to the framework/model/concept/ theory that the intervention was based on, and considers the mechanisms that led to various outcomes (both positive and negative). This is important in understanding how and why outcomes do or do not occur.

1) CPD intervention – included one criterion that assessed whether frameworks, models, or theories were used in explaining the problem, developing, and designing the intervention, and why the intervention was expected to work. This was included since quality evaluation is interwoven with design of interventions. For example, Bylund et al. [14] mentioned the Comskil model and that it is theory based, but they provided no detail about how this model related to the problem, or why the CPD intervention was expected to work.

2) Evaluation methods – included two criterion that focused on the description of the evaluation method chosen and inclusion of a rationale that justified the choice. This is important to show that the rationale for evaluation method is consistent with the selected evaluation methodology.
3) Measures – included four items that focused on the selected methods that captured not just the intended outcomes, but processes, unintended outcomes, and contextual factors, and that the rationale for the selection of measures was also included. This aligns with the calls to move to program evaluation approaches that capture more than just outcomes.
4) Analysis – included one criterion that focused on adequate description and justification of analysis as well as use of quantitative and qualitative methods to draw inferences from the data. Use of mixed methodology is important to address evaluation processes including the barriers and facilitators and unintended outcomes.
5) Results – included appropriate and detailed reporting of results. This ensures that the results are in line with the chosen measures and methods.
6) Discussion – included elaboration on associations between the intervention, outcomes, contextual factors, and unintended consequences. This would help determine how outcomes and unintended outcomes arose from the interplay between various intervention components and external elements.
