# Supplementary material for: Evaluation of Continuing Professional Development for Physicians – Time for Change: A Scoping Review
Source: Perspect Med Educ. 2023 Jun 2;12(1):198–207. doi: 10.5334/pme.838 (PMC10237247; doi:10.5334/pme.838)
Supplement: Appendix 3. — Table 4. Checklist for quality appraisal of Continuing Professional Development (CPD) program evaluations in individual studies. [file pme-12-1-838-s4.pdf]

APPENDIX 3

Table 4. Checklist for quality appraisal of Continuing Professional Development (CPD) program evaluations in individual studies.

Criteria	No	Partially	Yes	Scoring Criteria
**CPD Intervention Design**				
Use evidence or theory in explaining the design	39	44	18	In our scoring, a partial score was allocated when a study's intervention development and design was guided by the findings of previous research, and a score of 'yes' was given when a study used a formal framework/theory to elucidate strong links to the design and development of its CPD program and why it was expected to work.<br><br>*No – 0 = no elaboration*<br>*Partially – 1 = description of previous studies and how they have guided intervention development.*<br>*Yes – 2 = use of formal framework/theory (with strong links to the design, development and why it is expected to work).*
**Evaluation Methods**				
Evaluation model or framework clearly specified	89	2	10	*No – 0 = not specified.*<br>*Partially – 1 = mention of a model/framework/method but not clearly described.*<br>*Yes – 2 = clear description of method/model/framework used.*
Rationale for evaluation method	96	4	1	*No – 0 = no rationale provided.*<br>*Partially – 1 = limited rationale provided.*<br>*Yes – 2 = clear rationale provided.*
**Measures**				
Both processes and outcomes assessed	74	8	19	*No – 0 = only outcomes are assessed.*<br>*Partially – 1 = outcomes assessed; processes only incidentally brought up (not intentional).*<br>*Yes – 2 = both processes and outcomes are assessed.*
Description and rationale included for measures chosen	33	48	20	*No – 0 = No descriptions of measures and no rationales provided.*<br>*Partially – 1 = Limited or partial descriptions of measures and rationales without commenting on validity and/or reliability of the included measures.*<br>*Yes – 2 = Measures chosen for studying processes and/or outcomes of the intervention(s), including rationale for choosing them, their operational definitions, and their validity and reliability are included.*
Measures chosen allow unintended outcomes to be captured	71	0	30	*No – 0 = Measures did not allow for this, no qualitative measures selected.*<br>*Yes – 1= inclusion of qualitative or mixed-methods.*
Measures chosen allow contextual/external elements to be captured	62	8	31	*No – 0 = measures did not allow for capturing contextual elements included.*<br>*Partially – 1 = No qualitative measure, but measure aimed to account for this in quantitative analysis (e.g., inclusion in regression analysis).*<br>*Yes – 2 = questions included regarding potential contextual factors or allow for commenting on any potential contextual elements.*

**Analysis**				
Description and justification of analysis methods*	11	76	14	*No – 0 = analysis not clearly described, and no justifications provided.*<br>*Partially – 1 = only quantitative or qualitative analysis with poor descriptions and explanations performed.*<br>*Yes – 2 =inclusion of both quantitative and qualitative measures with detailed explanations.*
**Results**				
Outcomes reported with sufficient detail	9	28	64	*No – 0 = poor explanations of results.*<br>*Partially – 1 = results described with limited detail and more description would have been warranted.*<br>*Yes – 2 = clear explanation of results. If quantitative, it included 'p-values' and other relevant statistical information. If qualitative, it provided appropriate details based on selected methods (e.g., themes were well described, rather than mere inclusion of quotes without detailed explanations).*
Observed associations between outcomes, interventions, and relevant contextual/external elements	70	19	11	*No – 0 = no mention of contextual/external elements in the results and discussion sections.*<br>*Partially – 1 = commented on some contextual factors (e.g., experience, types of practice, management, etc.) but not intentionally measured them (incidental).*<br>*Yes – 2 = actively measuring contextual/external elements (e.g., barriers, resources, group differences).*
Unintended outcomes or consequences reported	92	0	9	*No – 0 = no reporting on unintended consequences.*<br>*Yes – 1 = Provided detailed descriptions of unintended consequences such as unexpected benefits, problems, failures, or costs associated with the CPD.*
**Discussion**				
Interpretations of results in context of theory in addition to current evidence included	11	87	3	*No – 0 = results were not linked to current evidence or theory.*<br>*Partially – 1 = either evidence or theory was used but there were poor explanations /linkages to findings.*<br>*Yes – 2 = general interpretations of the results in the context of current evidence and theory were provided.*
Consideration of the mechanism by which the intervention was intended to work (causal pathways) or alternative mechanisms or explanations?	49	44	8	*No – 0 = no explanations of potential mechanisms.*<br>*Partially – 1 = some explanations offered but not supported by theory.*<br>*Yes – 2 = the explanations were supported by theory.*

*0 = justification not reported, 1 = just qualitative or quantitative (or both but poorly described), 2 = both qualitative and quantitative (well described)
